# Extracardiac Vagal Stimulation-Assisted Cardioneuroablation: Dynamically Evaluating the Impact of Sequential Ganglionated Plexus Ablation on Vagal Control of SAN and AVN in Patients with Sinoatrial Node Dysfunction

**DOI:** 10.3390/jcdd9060188

**Published:** 2022-06-10

**Authors:** Weijie Chen, Zengzhang Liu, Peilin Xiao, Yanping Xu, Dan Li, Qingsong Xiong, Lili Zou, Fang Qin, Xiexin Tao, Junan Chen, Xianbin Lan, Huaan Du, Yuehui Yin, Zhiyu Ling

**Affiliations:** Department of Cardiology, The Second Affiliated Hospital of Chongqing Medical University, Chongqing 400010, China; cqmucwj@hospital.cqmu.edu.cn (W.C.); liuzengzhang666@163.com (Z.L.); xiaopeilin1982@163.com (P.X.); xuyanping@cqmu.edu.cn (Y.X.); lidan@stu.cqmu.edu.cn (D.L.); qingsongdr@163.com (Q.X.); 304230@hospital.cqmu.edu (L.Z.); qinfangheart@126.com (F.Q.); xenia.cqmu@gmail.com (X.T.); chenjunan91@163.com (J.C.); lanxb423@163.com (X.L.); duhuaan20@126.com (H.D.); yinyh63@163.com (Y.Y.)

**Keywords:** sinoatrial node dysfunction, cardioneuroablation, extracardiac vagal stimulation, cardiac ganglionated plexus

## Abstract

Cardioneuroablation (CNA) is proposed as a promising therapy for patients with sinoatrial node dysfunction (SND) that is mediated by excessive vagal tone. However, a series of urgent questions about CNA remain unanswered. From December 2020 to March 2022, six patients with symptomatic SND who underwent CNA were summarized in this report. Sequential CNA targeting Ao-SVC GP, PMLGP, RAGP, and LSGP was performed in patients, guided by fractionated intracardiac electrograms and dynamically evaluated by extracardiac vagal stimulation (ECVS). The results showed that Ao-SVC GP ablation led to a significant increase in heart rate (HR) and the elimination of sinus arrest evoked by ECVS, while the vagal responses of atrial ventricular block were eliminated by the ablation of PMLGP and LSGP. Post-procedure HR increased up to 64–86% of the maximum HR of an atropine test at baseline. The median HR from Holter monitoring increased from 52.8 ± 2.1 bpm at baseline to 73.0 ± 10.4 bpm after the procedure (*p* = 0.012) and to 71.3 ± 10.1 bpm at the six-month follow-up (*p* = 0.011). Bradycardia-related symptoms disappeared in all patients at the six-month follow-up. This case series reveals the feasibility of using the ECVS-assisted sequential CNA technique and indicates the critical role of ECVS in dynamically evaluating the impact of sequential CNA on the vagal control of SAN and AVN.

## 1. Introduction

The autonomic nervous system plays an important role in the physiological function of the cardiovascular system. An imbalance in autonomic tone is a critical pathophysiological mechanism in the development of cardiovascular disorders [1,2]. The roles of excessive activated sympathetic tone in hypertension, heart failure, and tachyarrhythmias have been systematically investigated and understood [1,2]. Nonetheless, the knowledge about and therapeutic strategies for the treatment of vagal tone-associated cardiovascular disorders are still limited.

The increased vagal tone mediated cardiovascular disorders in clinical practice mainly include functional sinoatrial node dysfunction (SND), functional atrioventricular block (AVB), and vasovagal syncope (especially the cardioinhibitory type and mixed type) [3,4,5,6]. Bradycardia-related symptoms are the most common clinical manifestation of these diseases, such as fatigue, dizziness, shortness of breath, and presyncope, which seriously affect the patients’ quality of life. Occasionally, some of these patients suffer from syncope and transient cardiac arrest [3,4,5,6]. Routine therapy for patients with severe bradycardia involves the implantation of a cardiac pacemaker. However, as a substantial portion of these patients are young, at ages <60 years or even <40 years, most of them refuse the pacemaker implantations. Out of question, the symptomatic patients who reject cardiac pacing therapy have to suffer and take the risk of syncope.

Excessive vagal tone is the root cause of symptomatic bradycardia in patients with functional SND, functional AVB, and vasovagal syncope [3,4,5,6]. Correspondingly, the efferent vagal nerve to the heart is a reliable therapeutic target for these patients with increased vagal tone. Furthermore, the cell bodies of the postganglionic neurons of cardiac-efferent vagal nerves have been demonstrated to be contained in the intrinsic cardiac ganglionated plexus (GP), which is embedded in the atrial wall and in epicardial fat pads [7,8]. Therefore, cardiac GP is proposed as a potential optimal interventional target for patients with symptomatic bradycardia that is induced by increased vagal tone. In 2005, the catheter-based ablation of the cardiac ganglionated plexus (cardioneuroablation, CNA) in patients with neurocardiogenic syncope, functional AVB, and SND, guided by a spectral analysis of local fractionated intracardiac electrograms to locate GPs was reported for the first time by Dr. Pachon et al. [9]. The results showed that the catheter ablation of cardiac GPs in 21 patients enrolled in that study effectively eliminated their bradycardia related symptoms during the 9.2 months follow up [9]. Nevertheless, the CNA technique that was used to identify the location of cardiac GPs via a spectral analysis of local fractionated intracardiac electrograms could only be performed using the customized software from Dr. Pachon’s study [9].

In recent years, the clinical research regarding the CNA technique has made remarkable progress. Purely anatomy-guided CNA [10,11] and intracardiac high-frequency stimulation (HFS)-guided CNA [12] were proposed and clinically performed in patients with symptomatic bradycardia that was induced by increased vagal tone. In 2016, Dr. Aksu et al. performed a simplified local fractionated intracardiac electrogram-guided CNA method without the assistance of specific spectral analysis software [13]. A number of small sample studies have also indicated the feasibility and efficacy of the local fractionated electrogram-guided CNA technique [14,15,16,17]. However, the research schemes of the CNA technique among published studies are varied. Regarding the target chamber for the CNA technique, the research groups of Dr. Pachon et al. [9,18] and Dr. Aksu et al. [16,17] ablated all of the atrial GP groups in both atria, while Dr. Yao et al. [12,19,20] mainly focused on the GP groups in the left atrium and Dr. Debruyne et al. [21,22] only ablated the GP groups in the right atrium. Additionally, the endpoint of the CNA procedure is also quite varied among published studies, including the disappearance of vagal responses that are evoked by extracardiac vagal stimulation (ECVS) [23]; the disappearance of vagal responses evoked by intracardiac HFS or radiofrequency (RF) application [12,24]; the elimination of intracardiac electrograms in targeted GP regions [9,13,16,17]; the elimination of atropine responses [25]; and improvements in the bradyarrhythmia or electrophysiological parameters [16,17]. In other words, a series of urgent questions related to the technical details of the CNA procedure have not been clearly illustrated, such as how to identify optimal candidates and GPs locations; what are the different impacts of sequential GP ablation on the vagal control of the sinoatrial node (SAN) and atrioventricular node (AVN); what are the ablation strategies for patients with different indications; what is the optimal endpoint of the CNA procedure; and what are the long-term outcomes of the CNA technique. Therefore, we conducted this case series and literature review to present the preliminary results of our experience with CNA, in order to summarize the current evidence regarding the application of CNA and investigate the answers to the abovementioned questions.

## 2. Methods

### 2.1. Study Population

From December 2020 to March 2022, patients with symptomatic bradyarrhythmia that was mediated by increased vagal tone were enrolled into this study. The detailed inclusion criteria were as follows: (1) patients with severe symptomatic bradycardia of functional SND or AVB (bradycardia was documented by electrocardiogram (ECG) and 24-h Holter monitoring and associated with excessive vagal tone with the evidence of correcting the bradycardia by a loading dose of atropine (0.04 mg/kg)), or patients with symptomatic vasovagal syncope (cardioinhibitory type or mixed type) documented by head-up tilt table testing (HUT); (2) aged between 14 and 65 years old; (3) no structural heart diseases; (4) rejected the option to implant a cardiac pacemaker. The exclusion criteria were as follows: (1) diagnosis of hypertension, diabetes, severe atherosclerosis, cerebrovascular disease, or organic diseases; (2) history of cardiac surgery and pacemaker implantation. The protocol was approved by our local ethics committee and performed in accordance with the Declaration of Helsinki. Written informed consent was obtained from all patients that were enrolled in the study.

### 2.2. Electrophysiological Study

All procedures were performed under general anesthesia. Atropine was required to be avoided during the induction and maintenance of anesthesia. Right ventricle pacing was necessarily performed to correct the severe bradycardia during the procedure. Bilateral femoral veins were punctured for the insertion of a quadripolar electrode catheter into the right ventricle, a decapolar steerable electrode catheter within the coronary sinus, another decapolar steerable electrode catheter within the right internal jugular vein at the level of the right upper wisdom tooth to perform ECVS, and a Thermocool^®^ SmartTouch (Biosense Webster, Diamond Bar, CA, USA) irrigated catheter in the right/left atrium for the ablation therapy. Surface ECG and intracardiac electrograms were continually monitored by a multichannel recording system during the whole procedure (LEAD-7000; JJET, Chengdu, China). Electrophysiological examinations were performed before and after every ablation of individual GP groups. Heart rate (HR), sinoatrial node recovery time (SNRT), Wenckebach cycle length (WBCL), atrial-His (AH) interval, and His-ventricle (HV) interval were all measured and collected during repeat electrophysiological examinations. The right atrium was targeted first and reconstructed using the 3D electroanatomic mapping system (CARTO3 Version 6; Biosense Webster, Diamond Bar, CA, USA). After the routine transseptal puncture, the left atrium was also reconstructed for further sequential GP ablation. Local fragmented atrial electrograms were evaluated for amplitude and number of deflections at filter settings of 30–500 Hz and a sweep speed of 200 mm/s. Unfractionated heparin was administrated intravenously at a bolus of 100 IU/Kg. Activated clotting time was measured every 30 min and maintained between 250 and 350 s.

### 2.3. Extracardiac Vagal Stimulation 

By directly activating the efferent vagal nerve to the heart, ECVS was proposed by Pachon et al. to be a useful method in evaluating the efficacy of the CNA technique at the end of the procedure [23,26]. At our center, ECVS was also used as a method for dynamically evaluating the impact of sequential GP ablation on the vagal control of SAN and AVN during the CNA procedure, which was very helpful in illustrating the variable role of individual GP groups in the vagal regulating mechanisms of SAN and AVN. Therefore, ECVS was repeatedly performed before and after every ablation of individual GP groups. The electrophysiological parameters and dynamic changes in vagal responses that were evoked by repeated ECVS were evaluated and recorded during the procedure. As proposed by Pachon et al. [23], ECVS was performed using a decapolar steerable electrode catheter within the right internal jugular vein at the level of the right upper wisdom tooth slightly toward the medial direction. The placement of this ECVS catheter to the level of the right upper wisdom tooth within the right internal jugular vein was guided by an X-ray fluoroscopy and assisted by internal jugular vein angiography if necessary. ECVS was delivered by a stimulator (SyNuo-C1; JJET, Chengdu, China) using the current mode with square wave pulses with 2 ms durations, frequencies of 30 Hz, and currents of 20 mA. The distal pair of electrodes of the decapolar steerable electrode catheter was used as the current output unit.

### 2.4. Mapping and Ablation of Cardiac GPs

After the anesthesia and puncture procedures, the 3D electroanatomic mapping of both atria were sequentially created using the Carto 3 system. The sinus atrial node, phrenic nerve, coronary sinus, and His bundle were labeled. According to the studies of Pachon et al. and Aksu et al., the identification of individual GPs locations was guided by the local fractionated intracardiac electrograms in the proposed anatomic distribution area in reconstructed bilateral atria [13,16,23,26]. The GPs were mapped and targeted in the following sequence at our center: the GP between the aortic root and the medial wall of the superior vena cava (Ao-SVC GP); the GP between the posterior wall of the coronary sinus ostium and the left atrium (PMLGP); the GP between the anterior antrum of the right superior pulmonary vein and the superior vena cava (RAGP); and the GP in the superolateral area around the root of the left superior pulmonary vein (LSGP). Published studies showed that the vagal control of SAN was mainly mediated by Ao-SVC GP and RAGP, while the vagal control of AVN contributed to PMLGP and LSGP [16,19,21,27]. Thus, at our center, we performed the CNA technique by sequentially targeting the Ao-SVC GP, PMLGP, RAGP, and LSGP. The radiofrequency ablation of GPs was performed in the power-controlled mode in a point-by-point fashion via the irrigated ST catheter. The energy was delivered at a power of 35 W, with an ablation index of 350–400 at the posterior wall and 450–500 in the remaining areas. After the ablation was completed for each GP group, ECVS and electrophysiological examination were repeatedly performed to evaluate the impact of sequential GP ablation on the vagal response and on the electrophysiological characteristics of the heart. Additionally, the atropine test was performed to assist in the evaluation after performing the whole sequential GP ablation procedure.

### 2.5. Endpoint Evaluation

The ablation endpoint in each GP group was the complete elimination of local fractionated intracardiac electrograms in the proposed anatomic distribution area of the GP. Moreover, the endpoints of the CNA procedure were achieved by the ablation in the proposed target GP groups (including Ao-SVC GP, PMLGP, RAGP, and LSGP), and the complete elimination of the vagal response that was evoked by repeated ECVS. If the vagal response evoked by repeated ECVS was not completely eliminated, additional ablation was performed to target the left and right inferior GP located at the inferior aspect of the posterior wall of the left atrium.

### 2.6. Follow Up

A follow-up of patients that were enrolled in the study was executed at discharge and at 1, 3 and 6 months after the CNA procedure. The follow-up involved clinical assessments, 12-lead ECGs, and 24-h Holter monitoring. Furthermore, the patients were also asked to record the related daily symptoms, such as fatigue, palpitations, dizziness, shortness of breath, presyncope, and syncope. All enrolled patients were prescribed novel oral anticoagulation agents for 2 months after the ablation procedure. The primary outcome was free from the severe bradycardia related symptoms of presyncope and syncope during the 6 months follow up.

### 2.7. Statistical Analysis

Continuous variables are presented as mean ± SD while the categorical variables are reported as count (percentage) of participants. Values related to PP interval, heart rate, AH interval, and HV interval were calculated based on the average of five-beat measurements, but not any single beat monitoring value, to attenuate the interferences of beat-to-beat variance. The differences of continuous variables among baseline and post-individual GP interventions were analyzed using a one-way ANOVA, followed by the necessary multiple comparisons with the Bonferroni test. However, if the homogeneity of the variances was violated, the differences of the variables were analyzed using Welch’s ANOVA, followed by a post hoc analysis with the Games–Howell test. A two-sided *p* < 0.05 was defined as being statistically significant. All statistical analyses were performed with SPSS statistical software (version23.0, Chicago, IL, USA).

## 3. Results

### 3.1. Patient Characteristics and Clinical Outcomes

From January 2020 to December 2021, six patients with functional SND who underwent CNA at our center were reported in this case series, while no patients with vasovagal syncope or functional AVB underwent the CNA treatment during this period. The clinical characteristics of these six patients are shown in Table 1. They suffered from severe bradycardia-related symptoms of syncope or presyncope and had positive response to the atropine test at baseline. According to the protocol, sequential ablations of Ao-SVC GP, PMLGP, RAGP, and LSGP guided by local fractionated intracardiac electrograms were performed on the patients and dynamically evaluated by the repeated ECVS technique. No vagal response was repeatedly induced by ECVS after the sequential ablation of GPs. At the same time, responses to the atropine test were negative at the end of the procedures. The patients’ post-procedure heart rates effectively increased up to approximately 64–86% of the maximum HR of the atropine test at baseline. The median HR from 24-h Holter monitoring increased significantly from 52.8 ± 2.1 bpm at baseline to 73.0 ± 10.4 bpm after the CNA procedure (*p* = 0.012) and to 71.3 ± 10.1 bpm at the 6-month follow-up (*p* = 0.011) (Table 2). The indicators of HR variability, including SDNN, RMSSD, and PNN50, decreased significantly (Table 2).

The disappearance of previous bradycardia-related symptoms was reported by all enrolled patients during their 6 to 12 months follow-up.

### 3.2. Vagal Response Evoked by ECVS during the Sequential CNA Procedure

**Figure 1:**
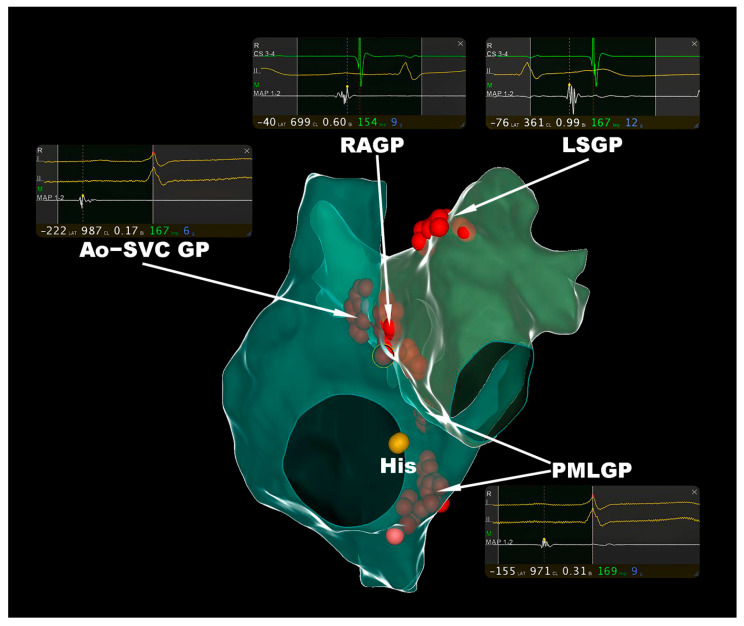
The representative figure of CNA procedure guided by local fractionated intracardial electrograms in proposed GP anatomic sites. The targeted fractionated intracardial electrograms of ablation catheter (MAP 1-2) from proposed GP sites (including Ao-SVC GP, PMLGP, RAGP, and LSGP) were respectively shown in the corresponding local electrogram windows. CNA = cardioneuroablation, GP = ganglionated plexus.

Guided by local fractionated intracardiac electrograms, the ECVS-assisted sequential CNA technique was successfully performed in all patients. A representative figure of the CNA procedure that was guided by local fractionated intracardial electrograms in the proposed GP anatomic sites at our center is shown in Figure 1. During the pre-ablation ECVS in the right internal jugular vein, sinus arrest was induced in all of the patients (Table 3). However, three patients suffered from complete cardiac arrest as no escape rhythm occurred, while the rest experienced a junctional escape rhythm that was secondary to the sinus arrest (Table 3). After the ablation of Ao-SVC GP, ECVS was repeatedly performed. Sinus responses, including sinus arrest and severe sinus bradycardia were not induced in all patients. Second- to third-degree AVB was induced in patients 1, 3, and 4, while the rest suffered from neither sinus responses nor AVB (Table 3). During the repeated ECVS after the sequential ablation of PMLGP, the previously induced AVB in patients 1, 3, and 4 was effectively improved, although patients 1 and 3 still suffered from second-degree AVB (Table 3).

**Figure 2:**
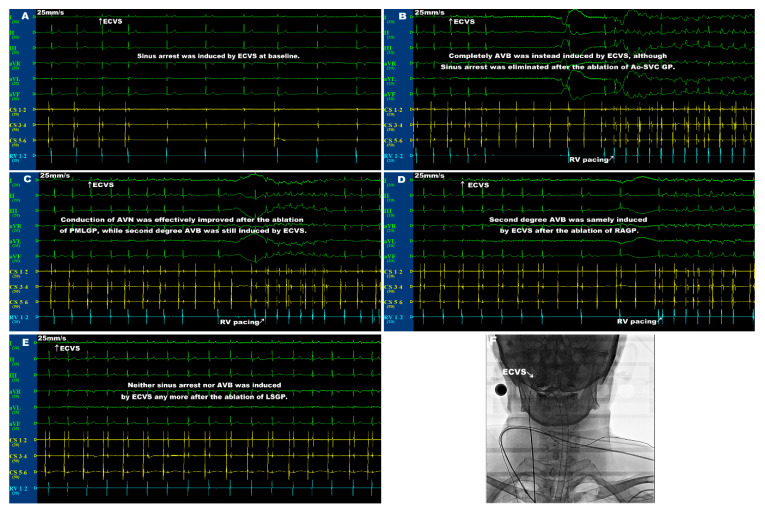
The representative figure of ECVS site and vagal responses of SAN and AVN during the sequential CNA procedure. (**A**) The vagal response of sinus arrest with a junctional escape rhythm was induced by ECVS at baseline. (**B**) After the ablation of Ao-SVC GP, the vagal response of sinus arrest evoked by ECVS was effectively eliminated, while complete AVB was instead induced. (**C**) After the sequential ablation of PMLGP, the previously induced complete AVB was effectively improved, although the patient still suffered from second-degree AVB. (**D**) After the further sequential ablation of RAGP, the second-degree AVB was samely induced by ECVS. (**E**) Neither sinus arrest nor AVB could be induced by ECVS any more after the further sequential ablation of LSGP. (**F**) X-ray fluoroscopy showed that ECVS was performed using a decapolar steerable electrode catheter within the right internal jugular vein at the level of the right upper wisdom tooth. ECVS = extracardiac vagal stimulation, SAN = sinoatrial node, AVN = atrioventricular node, CNA = cardioneuroablation, AVB = atrioventricular block, GP = ganglionated plexus.

However, after the sequential ablation of RAGP, second-degree AVB was repeatedly induced by ECVS in patients 1 and 3 without any improvement (Table 3). After the sequential ablation of LSGP, the second-degree AVB that was induced by ECVS in patients 1 and 3 was eliminated. Neither sinus response nor AVB were induced by ECVS in any patients (Table 3). Thus, the above data indicate that the ablation of Ao-SVC GP effectively eliminated the vagal response of SAN, while the vagal response of AVN was mainly eliminated by the ablation of PMLGP and LSGP. A representative figure of the ECVS site and the dynamic vagal responses of SAN and AVN during the sequential CNA procedure is presented in Figure 2.

### 3.3. Changes in Electrophysiological Parameters during the Procedure

**Figure 3:**
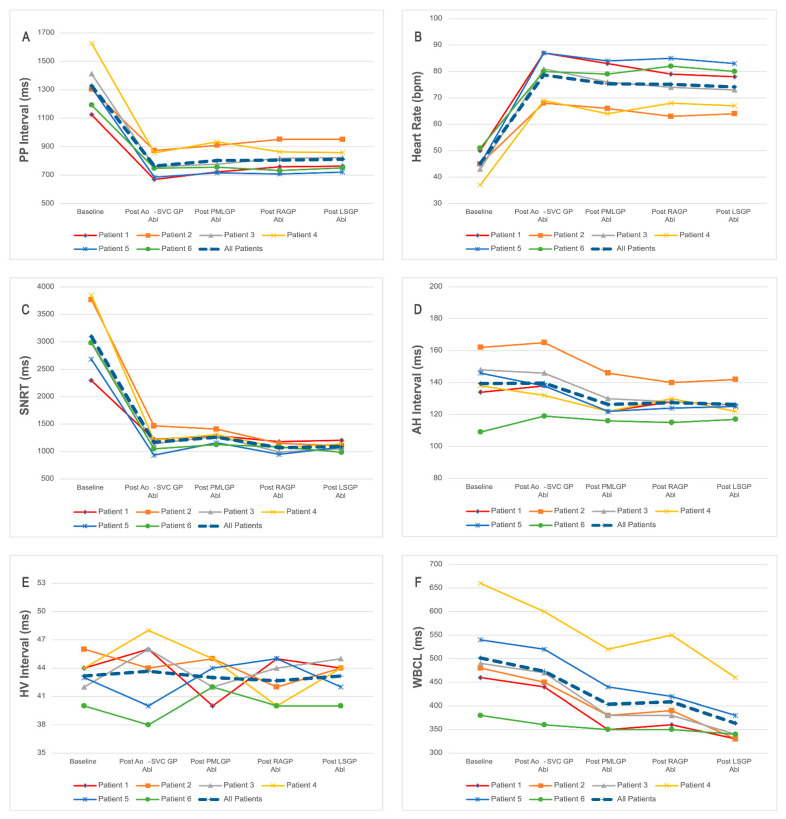
The dynamic changes of electrophysiological parameters of enrolled patients in the sequential CNA Procedure. (**A**,**C**) The PP internal and SNRT were significantly decreased by the ablation of Ao-SVC GP without further reduction during the sequential ablations of PMLGP, RAGP, and LSGP. (**B**) The heart rate was significantly increased by the ablation of Ao-SVC GP without further increasing during the sequential ablations of PMLGP, RAGP, and LSGP. (**D**,**F**) The AH interval and WBCL were obviously decreased by the ablation of PMLGP. (**E**) The HV interval did not show any significant changes during the sequential CNA procedure. CNA = cardioneuroablation, PP = p waves, GP = ganglionated plexus, Abl = ablation, SNRT = sinoatrial node recovery time, AH = atrial-His, HV = His-ventricle, WBCL = Wenckebach cycle length.

The influences of sequential ablations of Ao-SVC GP, PMLGP, RAGP, and LSGP on the electrophysiological characteristics of the heart are summarized in Table 4 and Figure 3. As shown, PP internal was significantly decreased by the ablation of Ao-SVC GP (*p* = 0.002) without further reduction during the sequential ablations of PMLGP, RAGP, and LSGP. Correspondingly, heart rate was also significantly increased by the ablation of Ao-SVC GP (*p* = 0.001) without further increasing during the following ablations of PMLGP, RAGP, and LSGP. Simultaneously, the SNRT was remarkably decreased by the ablation of Ao-SVC GP (*p* = 0.003) without further reductions during the sequential ablations of PMLGP, RAGP, and LSGP. The AH interval was significantly decreased by the ablation of PMLGP (*p* = 0.025) without corresponding reductions during the ablation of Ao-SVC GP, RAGP, and LSGP. The HV interval did not show any significant changes during the sequential CNA procedure. The WBCL interval was remarkably decreased by the sequential GP ablation procedure (*p* < 0.001). However, only the ablation of PMLGP induced a statistical decrease in the WBCL interval (*p* = 0.024), while no statistical changes were found for the WBCL interval during the ablation of Ao-SVC GP, RAGP, and LSGP. Thus, the sinus rate and SNRT were mainly improved by the ablation of Ao-SVC GP, and the atrial ventricular conduction that was evaluated by the AH interval and WBCL interval was significantly improved by the ablation of PMLGP.

## 4. Discussions

This case series indicates that ECVS can stably induce the cardioinhibitory vagal responses of sinus arrest or atrial ventricular block by activating the efferent vagal nerve to the heart, which is very helpful in dynamically evaluating the different influences of individual GP ablations on the vagal responses and electrophysiological characteristics of SAN and AVN during the CNA procedure. The ablation of Ao-SVC GP led to a significant increase in sinus heart rate and the effective elimination of the vagal response of sinus arrest evoked by ECVS, while the vagal response of atrial ventricular block was effectively eliminated by the ablations of PMLGP and LSGP. Concurrently, the ablation of PMLGP induced the statistical reductions in the AH interval and WBCL interval. However, the ablation of RAGP had no effects on the vagal response of atrial ventricular block that was evoked by ECVS. Finally, the bradycardia-related symptoms of these six patients were eliminated during the 6 to 12 months follow-up after the ECVS-assisted can technique was performed.

Recent advances in anatomic studies redemonstrated that the postganglionic neurons of efferent vagal nerves to the heart within the cardiac GPs were mainly embedded in the atrial wall and in the nearby epicardial fat pads in mammals and humans [7,8]. A catheter-based CNA technique in the endocardium had been proposed to effectively decrease the overactivated cardiac vagal tone by directly damaging the postganglionic neurons of the efferent vagal nerves to the heart within cardiac GPs [9,12,18]. Furthermore, excessive efferent vagal tone to the heart is the root cause of symptomatic bradycardia in patients with functional SND, functional AVB, and vasovagal syncope (especially cardioinhibitory type and mixed type) [3,4,5,6]. Correspondingly, a catheter-based CNA technique is proposed as a critical non-drug treatment for relieving the bradycardia-related symptoms in patients with SND, functional AVB, and vasovagal syncope [9,10,12,16].

However, the first key issue of the CNA technique is how to screen the real beneficiaries in proposed candidates with vagal tone medicated symptomatic bradyarrhythmia. The main purpose of the screening procedure is to evaluate whether the symptomatic bradyarrhythmia in proposed candidates is definitely induced by the overactivated cardiac vagal tone. The elimination of cardiac vagal tone resulting in the correction of bradyarrhythmia indicates the root cause of a patient’s excessive vagal tone and identifies the potential candidates of the CNA technique. Thus, through successfully correcting bradyarrhythmia by temporarily eliminating the vagal tone, an atropine test has been used as the main method to screen appropriate candidates of the CNA technique in this case series and in published studies [9,18,23,25,28]. On the contrary, increasing the efferent vagal tone to the heart by a head-up tilt test probably induces severe bradycardia-related events in these patients. Correspondingly, a head-up tilt test was also used to assist in screening the appropriate candidates of the CNA technique [9,12,18,23,29]. Moreover, as shown in this case series, the GP locating in the CNA procedure was guided by local fractionated intracardiac electrograms. To attenuate the interferences because of atrial fibrosis, older patients, and candidates with long-term chronic diseases, such as hypertension, diabetes, severe atherosclerosis, structural heart diseases, pulmonary illnesses, and thyroid dysfunctions, should be excluded [9,10,13,18,23].

During the CNA procedure, how to locate targeted GP sites in the atrium is also a key issue to be discussed. As shown in this case series, the locations of GP sites at our center were guided by the local fractionated intracardial electrograms, a technique firstly reported by Dr. Pachon et al. [9,18,23]. According to the research of Dr. Pachon et al., compared with the normal intracardial electrograms of a compacted myocardium, the distribution of neurons within GPs that are embedded in the atrial wall interferes with local atrial electrical conduction, which results in the fractionated intracardial electrograms [9,18,23]. Thus, fractionated intracardial electrogram in the potential anatomical distribution area of GP was proposed to guide us to locate the GP sites during the CNA procedure. The local fractionated intracardial electrogram-guided CNA technique was widely applied by Dr. Aksu et al. in young patients with excessive vagal tone medicated bradyarrhythmia in recent years [13,14,15,16,30]. Similar to the present study, previously published studies of Dr. Aksu et al. also demonstrated the feasibility and efficacy of the fractionated intracardial electrogram-guided CNA technique [13,14,15,16,30]. Additionally, locating GP sites based on anatomical criteria was applied by Dr. Qin et al. [10] in their CNA research on patients with sinus bradycardia, which showed the primary feasibility of the CNA procedure guided by an anatomical approach. However, the anatomical distribution area of GP sites was not consistent among patients. Although the CNA technique guided by anatomical approach could partially damage GPs, the accuracy and targeting are not guaranteed. Moreover, the vagal response that was evoked by endocardial HFS was also used to locate the distribution of GPs in a series of published papers [12,13,14,19]. Nevertheless, the vagal response that was evoked by endocardial HFS was frequently found in the distribution of LSGP but rarely in Ao-SVC GP, while the ablations of Ao-SVC GP in this case series also led to the definite CNA effects [12,31]. In other words, locating GP sites via vagal responses that were evoked by endocardial HFS provided higher specificity but less sensitivity. Furthermore, the irregular RR interval of atrial fibrillation commonly induced by endocardial HFS also interfered with the judgement of vagal response [12,24]. Based on the above discussions, the local fractionated intracardial electrogram-guided CNA technique is repeatedly supported by recent evidence and can be easily applied to clinical practice. Additionally, cardiac imaging techniques, including computed tomography [32] and magnetic resonance imaging [33] had been used to reconstruct the epicardial fat pads containing the cardiac GPs. The reconstructed epicardial fat pads can be merged with the 3D electroanatomic model of the atrium in the CNA procedure to potentially help the rapid locating of the local fractionated intracardiac electrograms in future clinical studies. Moreover, the feasibility of radionuclide imaging with SPECT to non-invasively identify the location of cardiac GPs was reported by Dr. Stirrup in 2020 [34], which may have better application prospects in locating GPs during the CNA procedure in future.

Furthermore, the number of GP site groups that should be ablated in the CNA procedure is also an urgent question that needs to be answered. Dr. Yao et al. mainly ablated the groups of GP sites in the left atrium in the CNA procedure [12,20], while Dr. Debruyne et al. only targeted the groups of GP sites in the right atrium [21,22]. Published studies also indicated the potential feasibility of the CNA procedure only focusing on the ablation of RAGP or Ao-SVC GP [21,35]. Concurrently, groups of GP sites in both the left atrium and the right atrium were ablated by Dr. Pachon et al. [9,18,23] and Dr. Aksu et al. [13,15,16] during their CNA procedures. However, the anatomical studies indicated that interganglionic communication definitely existed among the groups of the cardiac GP sites [7,27,31,36]. As shown in this case series and previously published studies, both SAN and AVN were simultaneously innervated and regulated by multiple groups of GP sites [27,31,37]. Although the vagal responses of SAN that were evoked by ECVS in all the enrolled patients of this case series were eliminated by the ablation of Ao-SVC GP, the vagal responses of AVN that were evoked by ECVS in patients 1 and 3 were eliminated by the ablations of PMLGP and LSGP. As indicated in patients 1 and 3, the ablation of RAGP had no effects on eliminating the vagal responses of AVN evoked by ECVS. In other words, although interganglionic communications exist among GPs, the vagus nerve-regulating mechanism that was mediated by various GP groups between SAN and AVN were completely different. By dynamically evaluating the vagal responses of SAN and AVN to repeated ECVS during the sequential CNA procedure, this case series indicated that the vagal regulation of SAN was mainly mediated by Ao-SVC GP, while the vagal regulation of AVN was mainly mediated by PMLGP and LSGP. Thus, the can procedure focusing on a single group of GP sites is insufficient, while a single atrial chamber-focused CNA technique is also inaccurate. Investigating and clarifying the different vagus nerve-regulating mechanisms of SAN and AVN mediated by the cardiac GPs are critical for determining the appropriate targeted GP sites in the CNA procedures. Moreover, based on the different primary diagnoses of functional SND, functional AVB, or cardioinhibitory vasovagal syncope, similar to the present study, repeated ECVS to activate the efferent vagal nerve to the heart can dynamically evaluate the impact of sequential GP ablation on the vagal control of SAN and AVN and can probably guide the achievements of personalized CNA protocols in different potential candidates.

Moreover, how to define the ideal endpoints is another urgent question that needs to be answered in the CNA procedure. Dr. Yao et al. [12,24] used the elimination of the vagal response that was evoked by endocardial HFS after the ablation of GP sites as the endpoints of the CNA procedure. The elimination of the vagal response that is evoked by endocardial HFS can be proposed as an indicator of effective damage to the targeted GP sites. However, considering the endpoint of the CNA procedure to be the elimination of the vagal response that is evoked by endocardial HFS does not answer the critical question of whether the patient will suffer from a symptomatic vagal response again when the efferent vagal nerve to the heart is overactivated outside the hospital, similar to the efferent vagal nerve that is activated by ECVS in present study. Thus, taking the elimination of the vagal response that is evoked by endocardial HFS as an endpoint of the CNA procedure has its limitations. Eliminating the intracardiac fractionated electrograms in all proposed anatomical GP site distribution areas was considered as an endpoint of the CNA procedure by Dr. Aksu et al. [13,16,30,38,39]. Similarly, the critical question of whether the patient will suffer from symptomatic vagal response again after discharge from the hospital following the CNA procedure remains unanswered. Nevertheless, eliminating the intracardiac fractionated electrograms in the proposed anatomical GP site distribution areas was an indicator regarding the effective ablation lesions that were produced in the proposed GP sites. Improvements in the electrophysiological parameters, such as an increase in heart rate and shortening of the PR interval were also proposed to be endpoints of the CNA procedure in some published papers [16,17]. However, as shown in this case series, heart rate was significantly increased just after the ablation of Ao-SVC GP, while the vagal response of significant AVB was still stably induced by activating the efferent vagal nerve to the heart via ECVS in patients 1, 3 and 4. Thus, without checking the vagal response that is evoked by activating the efferent vagal nerve to the heart, improvements in the electrophysiological parameters cannot directly indicate the final success of the CNA procedure. At the early stage of the CNA study, failure of the atropine test after GP ablation was defined as the endpoint of the CNA procedure [25]. As shown in this case series, the atropine test was negative in all enrolled patients after the CNA procedure, while which was positive during the screening procedure. Noticeably, compared with the level at baseline, their heart rates were significantly increased after the CNA procedure, which definitely disturbed the patients’ heart response to atropine. Thus, taking the failure of the atropine test as endpoints of the CNA procedure also presents limitations. Comprehensively, the ideal endpoint of the CNA procedure should indicate that the risk of suffering from symptomatic vagal response is eliminated when the efferent vagal nerve to the heart is overactivated outside the hospital. Thus, as shown in this case series, the total disappearance of the vagal responses that are evoked by ECVS during the CNA procedure is direct evidence indicating the elimination of the cardioinhibitory vagal responses when the cardiac vagal tone is overactivated by ECVS. Correspondingly, the elimination of the vagal responses that are evoked by ECVS during a CNA procedure can be taken as the ideal endpoint of the CNA technique.

Therefore, based on the above discussions, recent advances in neural anatomic studies repeatedly provide a sound theoretical basis for the application of a catheter-based CNA technique for treating patients with symptomatic bradyarrhythmia that is mediated by excessive cardiac vagal tone, through directly damaging the postganglionic neurons of the efferent vagal nerves to the heart within the cardiac GPs. Besides assisting in the dynamic evaluation of the impacts of sequential GP ablation on the vagal control of SAN and AVN, repeated ECVS during a CNA procedure is also critical in determining whether the endpoint of the CNA procedure is reached. The results of this case series indicated that the ablation of Ao-SVC GP led to a significant decrease in the vagal control of SAN, while the vagal control of AVN was significantly diminished by the ablations of PMLGP and LSGP. Meanwhile, the ablation of RAGP had no effects on the vagal control of AVN. Although remarkable progress in the CNA technique has been made in recent years, the present study is still the first report to dynamically evaluate the impact of sequential GP ablation on the vagal control of SAN and AVN via repeated ECVS. However, some noteworthy limitations of the present study must be pointed out. Firstly, this case series only analyzed and reported on the primary results of six enrolled patients at our center. Secondly, the sequential CNA procedure was only performed in the order of Ao-SVC GP, PMLGP, RAGP, and LSGP in the present study. The prior GP ablation will potentially disturb the evaluation of the effects following GP ablation on the vagal control of SAN and AVN. Thus, sequential CNA by targeting the GP groups in a different order should be investigated in future clinical studies. Moreover, the vagal response to ECVS at baseline was only evaluated in sinus rhythm. The induced sinus arrest and junctional rhythm by ECVS probably concealed the baseline vagal response of AVN to ECVS, which perhaps masked the effects of Ao-SVC GP ablation on the vagal control of AVN in the present study. Atrial pacing during ECVS at baseline should be performed to reveal the potential role of Ao-SVC GP ablation on AVN in future study protocols. Therefore, the results that were reported and the key questions that were discussed above need to be urgently confirmed and answered by large-scale, multi-center, randomized, controlled clinical trials in the near future.

## 5. Conclusions

Besides assisting in the dynamic evaluation of the effects of sequential GP ablation on the vagal control of SAN and AVN, repeated ECVS during a CNA procedure is also critical in determining whether the endpoint of the CNA procedure is reached. The primary results of this case series indicated that the ablation of Ao-SVC GP mainly led to a significant decrease in the vagal control of SAN, while the vagal control of AVN was significantly diminished by the ablations of PMLGP and LSGP. By involving the Ao-SVC GP, PMLGP, RAGP, and LSGP, the ECVS-assisted CNA technique significantly increases the heart rate of patients with symptomatic SND that is mediated by excessive vagal tone, accompanying the successful relief of bradycardia-related symptoms during the 6 to 12 months follow-up.

## Figures and Tables

**Table 1:** The clinical characteristics of enrolled patients.

Pts	Sex	Age (yrs)	BMI (kg/m^2^)	DIAG	DUR (yrs)	CC	Achieved Endpoints of Procedure	Atropine Test Pre-Ablation				HR Post Abl (bpm)	Atropine Test Post-Abl (+/−)	HR Post Abl/Max. HR in Atropine Test Pre-Abl (%)	Follow-Up	
								Basic HR (bpm)	Max. HR (bpm)	HR Increase (%)	Result (+/−)				DUR (Ms)	Related Symptoms
Pt 1	F	55	23.1	SND	20+	fatigue, dizziness, presyncope	(1) Complete the ablation of Ao-SVC GP, PMLGP, RAGP, and LSGP; (2) No vagal response induced by ECVS.	52	121	132	(+)	78	(−)	64	6	Disappeared, no presyncope /syncope
Pt 2	F	50	22.0	SND	20+	fatigue, dizziness, syncope		46	80	74	(+)	66	(−)	83	6	Disappeared, no presyncope /syncope
Pt 3	F	53	22.8	SND	10+	fatigue, dizziness, presyncope		53	97	83	(+)	74	(−)	76	9	Disappeared, no presyncope/syncope
Pt 4	M	56	24.8	SND	10+	fatigue, palpitation, syncope		54	90	67	(+)	69	(−)	77	12	Disappeared, no presyncope/syncope
Pt 5	M	19	23.9	SND	6	fatigue, palpitation, syncope		49	102	108	(+)	84	(−)	82	6	Disappeared, no presyncope/syncope
Pt 6	F	43	20.4	SND	3	fatigue, palpitation, syncope		50	94	88	(+)	81	(−)	86	12	Disappeared, no presyncope/syncope

Pts = patients, F = female, M = male, DIAG = diagnosis, SND = sinoatrial node dysfunction, yrs = years, DUR = duration, CC = chief complaint, ECVS = extracardiac vagal stimulation, HR = heart rate, Max. = maximal, Abl = ablation, Ms = months. See text for detailed description.

**Table 2:** The 24-h Holter monitoring results of enrolled patients.

Items	Baseline	Before Discharge	6 Months	*p* Value for One-Way ANOVA
Minimal HR (bpm)	34.2 ± 4.0	54.3 ± 12.5 *	52.0 ± 8.4 *	*p* = 0.002
Median HR (bpm)	52.8 ± 2.1	73.0 ± 10.4 *	71.3 ± 10.1 *	*p* < 0.001
Maximal HR (bpm)	107.7 ± 23.6	103.7 ± 10.7	111.7 ± 18.0	*p* = 0.550
SDNN (ms)	180.3 ± 56.5	80.2 ± 31.4 *	86.2 ± 29.5 *	*p* = 0.009
RMSSD (ms)	57.3 ± 11.7	30.3 ± 25.5 *	28.0 ± 15.9 *	*p* = 0.004
PNN50 (%)	27.5 ± 8.3	6.7 ± 5.9 *	5.5 ± 3.6 *	*p* < 0.001

* *p* < 0.05 versus baseline. Statistics was conducted using one-way ANOVA. HR = heart rate, SDNN = standard deviation of normal-to-normal intervals, RMSSD = root mean square of successive RR interval differences, PNN50 = percentage of successive RR intervals that differ by >50 ms. See text for detailed description.

**Table 3:** The vagal responses of SAN and AVN evoked by repeated ECVS during the sequential CNA procedure of enrolled patients.

Patients	Baseline	Post Ao-SVC GP Abl	Post PMLGP Abl	Post RAGP Abl	Post LSGP Abl
Patient 1	Sinus arrest without escape rhythm (complete cardiac arrest)	No sinus response, but high degree AVB	No sinus response, but type I 2nd AVB	No sinus response, but type I 2nd AVB	No sinus response, No AVB
Patient 2	Sinus arrest with junctional escape	No sinus response, No AVB	No sinus response, No AVB	No sinus response, No AVB	No sinus response, No AVB
Patient 3	Sinus arrest with junctional escape	No sinus response, but III-degree AVB	No sinus response, but type II 2nd AVB	No sinus response, but type II 2nd AVB	No sinus response, No AVB
Patient 4	Sinus arrest without escape rhythm (complete cardiac arrest)	No sinus response, but type II 2nd AVB	No sinus response, No AVB	No sinus response, No AVB	No sinus response, No AVB
Patient 5	Sinus arrest with junctional escape	No sinus response, No AVB	No sinus response, No AVB	No sinus response, No AVB	No sinus response, No AVB
Patient 6	Sinus arrest without escape rhythm (complete cardiac arrest)	No sinus response, No AVB	No sinus response, No AVB	No sinus response, No AVB	No sinus response, No AVB

SAN = sinoatrial node, AVN = atrioventricular node, ECVS = extracardiac vagal stimulation, CNA = cardioneuroablation, GP = ganglionated plexus, Abl = ablation, AVB = atrioventricular block. See text for detailed description.

**Table 4:** The statistical results of electrophysiological parameters during the sequential CNA procedure.

Items	Baseline	Post Ao-SVC GP Abl	Post PMLGP Abl	Post RAGP Abl	Post LSGP Abl	*p* Value for One-Way ANOVA
PP interval (ms)	1329.3 *±* 177.4	765.0 *±* 84.5 *	802.5 *±* 95.5 *	805.5 *±* 91.8 *	811.7 *±* 84.8 *	*p* < 0.001
HR (bpm)	45.2 *±* 5.1	78.7 *±* 8.4 *	75.3 *±* 8.5 *	75.2 *±* 8.5 *	74.2 *±* 7.5 *	*p* < 0.001
SNRT (ms)	3095.5 *±* 608.6	1170.2 *±* 181.6 *	1262.8 *±* 103.9 *	1070.5 *±* 88.8 *	1094.8 *±* 75.3 *	*p* < 0.001
AH interval (ms)	139.5 *±* 17.8	139.7 *±* 15.3	126.3 *±* 10.6 #	127.5 *±* 8.1	126.3 *±* 8.4	*p* = 0.01
HV interval (ms)	43.2 *±* 2.0	43.7 *±* 3.9	43.0 *±* 2.0	42.7 *±* 2.3	43.2 *±* 1.8	*p* = 0.95
WBCL (ms)	501.7 *±* 93.5	473.3 *±* 80.9	403.3 *±* 65.9 *,#	408.3 *±* 73.6 *,#	363.3 *±* 50.9 *,#	*p* < 0.001

* *p* < 0.05 versus baseline, # *p* < 0.05 versus post-Ao-SVC GP Abl. Statistics were conducted using one-way ANOVA followed by multiple comparisons with Bonferroni test. CNA = cardioneuroablation, GP = ganglionated plexus, PP = p waves, HR = heart rate, SNRT = sinoatrial node recovery time, AH = atrial-His, HV = His-ventricle, WBCL = Wenckebach cycle length, Abl = ablation. See text for detailed description.

## Data Availability

Data are available from the corresponding author upon reasonable request.

## Nonstandard Abbreviations and Acronyms

AH	atrial-His
AVB	atrioventricular block
AVN	atrioventricular node
CAN	cardioneuroablation
ECG	electrocardiogram
ECVS	extracardiac vagal stimulation
GP	ganglionated plexus
HFS	high-frequency stimulation
HR	heart rate
HUT	head-up tilt table testing
HV	His-ventricle
PNN50	percentage of successive RR intervals that differ by > 50ms
RF	radiofrequency
RMSSD	root mean square of successive RR interval differences
SAN	sinoatrial node
SDNN	standard deviation of normal-to-normal intervals
SND	sinoatrial node dysfunction
SNRT	sinoatrial node recovery time
WBCL	Wenckebach cycle length
Ao-SVC GP	GP between the aortic root and the medial wall of the superior vena cava
PMLGP	GP between the posterior wall of the coronary sinus ostium and the left atrium
RAGP	GP between the anterior antrum of the right superior pulmonary vein and the superior vena cava
LSGP	GP in the superolateral area around the root of the left superior pulmonary vein
